
JOURNAL INFORMATION
==============================
NLM Title Abbreviation: Buffalo Med J
NLM Journal ID: 0027534

Buffalo Medical Journal

ISSN: 1040-3817
Publisher: Biomedical Journal Digitization Project

ARTICLE INFORMATION
==============================
PMCID: PMC8735640
PMID: 36886963
Article version: 1
Subjects: Items

Items

Print publication date: 1906 May
Volume: 61
Issue: 10
First page: 630
Last page: 630
License: This work is free of known copyright restrictions. For more information, please see PMC Back Issue Digitization.
License URL: https://creativecommons.org/publicdomain/mark/1.0/

==============================

### ITEMS.

We learn from Messrs. Kress & Owen Co., of New York, that they promptly resigned their membership in the Proprietary Association of America when it became apparent that this association was antagonistic to the medical profession. Since the commencement of their business, some ten years ago, they have advertised Glyco-Thymoline only to the profession by clinical reports, and through medical journals, and have no intention of changing their methods of doing business.—*Journal of the Association of Military Surgeons.*

---

Mr. William T. Collier, whose card appears in the Journal, has been in the medical book business in Buffalo for the past thirty years. He can obtain any medical book published, on short notice. See him for medical books.

---

The Lyman D. Morse Advertising Agency, which has been in business at 38 Park Row, New York, for many years, changed its name April 1, 1906, to the Morse International Agency. It began business in 1849, having been established by S. M. Pettengill, and continued under various changes of partners until Mr. Morse took over the business in his own name about 1895. It will easily be seen that it is the oldest advertising establishment in the country and its enlarged sphere of operation has justified the change of name alluded to. Owing to increase of business, necessitating larger offices, the corporation removed its offices on May 1 to the Revillion Building, 19 West 34th Street, New York.

---

**FOR SALE**—Two new Static Electric Machines, for less than cost of manufacture, apply to C. B. Rowell, M. D., 898 Niagara Street, Buffalo.
